# Experiences and insights from parents of children with temporary feeding tubes

**DOI:** 10.1177/13674935251370415

**Published:** 2025-08-23

**Authors:** Claire Reilly, Jeanne Marshall, Rebecca Packer, Nikhil Thapar, Maryanne Syrmis, Nadine Frederiksen, Kristie L Bell

**Affiliations:** 1 School of Health & Rehabilitation Sciences, 1974 The University of Queensland, Brisbane, QLD, Australia; 2 Queensland Children’s Hospital, Children’s Health Queensland Hospital and Health Service, South Brisbane, QLD, Australia; 3 Gastroenterology, Hepatology and Liver Transplant, 67568 Queensland Children’s Hospital (QCH), Brisbane, QLD, Australia; 4 Faculty of Health, School of Exercise & Nutrition Sciences, Queensland University of Technology, Brisbane, QLD, Australia; 5 School of Medicine, 1974 The University of Queensland, Brisbane, QLD, Australia

**Keywords:** Child, enteral nutrition, infant, parents, questionnaires, surveys

## Abstract

This study aimed to describe parents’ experiences of children with temporary feeding tubes by surveying parents whose children required tubes. This survey included three sections including; (A) Depression Anxiety Stress Scale (DASS-21); (B) Paediatric Assessment Scale for Severe Feeding Problems (PASSFP); and (C) open-ended questions on satisfaction and experiences. Data were analysed using descriptive statistics and inductive content analysis. A total of 44 parent participants completed the survey. Most participants reported satisfaction with their child’s care, although for most, tube removal occurred after ≤5 days (n = 25, 57%). Children who were discharged home with a temporary feeding tube (n = 7, 16%) had more feeding difficulties. Two themes were identified: 1) navigating tube feeding and 2) health service experiences. Although parents were generally positive about their child’s care, some described ongoing medical and psychosocial impacts. Many parents desired more involvement in decision-making and tube feeding care. Parents described varied experiences regarding temporary tube feeding both in hospital and after discharge home. Further studies are needed across all spectrums of temporary tube feeding care, especially those discharged home. These findings underscore a need for enhanced support and education for parents, which could improve outcomes for children with temporary feeding tubes.

## Introduction

Hospitalised children often require temporary feeding tubes (e.g. nasogastric tubes, nasojejunal tubes, orogastric tubes), to support growth and nutrition when oral intake is insufficient (Lyman et al., 2016). A feeding tube is typically defined as being temporary if required for less than 4 weeks (Braegger et al., 2010). However, the duration of ‘temporary’ tube feeding in the literature varies widely, ranging from weeks to years (Reilly et al., 2023). There have been an increasing number of children discharged home from hospital with temporary feeding tubes (Rosen et al., 2016). Side effects of temporary feeding tubes are well recognised and can include pain, nasal irritation, and risk of aspiration (Krom et al., 2019; Syrmis et al., 2024). Long-term side effects of temporary feeding tubes can include oral aversion and tube feeding dependency, which can have significant developmental and psychosocial impacts on children and their parents (Dunitz-Scheer et al., 2009). Unfortunately, little is understood about how to prevent these negative outcomes for children and their families.

In contrast to temporary feeding tubes, gastrostomy tubes are typically required for children who require prolonged nutrition support and are generally considered long-term or permanent (Heuschkel et al., 2015). The experiences of parents caring for children with gastrostomy tubes, have been more extensively studied than temporary feeding tubes, with research describing both negative outcomes, such as tube-related complications and difficulties managing at home, and positive outcomes such as improved quality of life (Avitsland et al., 2012; Brotherton et al., 2007; Sullivan et al., 2004). Unfortunately, these findings cannot be directly compared to experiences of children with temporary feeding tubes. Similarly, research studies that do not differentiate between types of feeding tubes (e.g. nasogastric or gastrostomy tubes) on quality of life or family experiences cannot provide an accurate representation for children with temporary feeding tubes (Calderon et al., 2011; Coghlan, 2018; Dadich et al., 2023; Ferguson and Campbell, 2014; Hopwood et al., 2020; Pahsini et al., 2016; Pedersen et al., 2004; Serjeant and Tighe, 2022; Wilken, 2012). Thus, experiences and well-being of children with temporary feeding tubes remain largely unknown, representing a notable gap in existing research. Without understanding their experiences, these children and their families are unlikely to receive the necessary care and support.

Challenges associated with temporary feeding tubes have been acknowledged. The European Society for Paediatric Gastroenterology Hepatology and Nutrition (ESPGHAN) Comittee on Nutrition noted a high rate of discomfort caused by temporary feeding tubes for children, suggesting that gastrostomy tubes are a preferable option longer-term (Braegger et al., 2010). Similarly, children with temporary feeding tubes for long periods of time can experience nasal discomfort (El-Matary, 2008), however some research still recommends prolonged temporary tube feeding in children (Berman et al., 2022). In a British study, children reported severe pain from their temporary tube feeding insertions despite their parents and health professionals rating their pain as less severe (Crellin and Johnston, 2005). Therefore, understanding healthcare needs, experiences, and outcomes for children with temporary feeding tubes and their families is essential to improving care.

Temporary tube feeding involves three phases: planning, maintenance, and weaning (Dunitz-Scheer et al., 2009). Emerging research explores parental experiences during tube weaning phase (Cipolla et al., 2022; Edwards et al., 2016; Lively et al., 2023), but no known research explores parental experiences from planning/placement to removal. Lively and colleagues interviewed 14 parents on their experiences about their child transitioning from tube to oral feeding after a tube weaning program (Lively et al., 2023). Seven themes were identified through inductive thematic analysis acknowledging stressful, yet transformative journeys parents experienced during their child’s tube wean. Findings from this study highlighted parental desire for involvement in decision-making of their child’s feeding tube. However, this research focused predominantly on tube removal and did not differentiate between types of feeding tubes children had (Lively et al., 2023). Consequently, current evidence lacks elucidation of key issues faced by families of children with temporary feeding tubes. To better support these children and their families, we must understand their experiences from the time of tube insertion.

## Aim

To describe experiences of parents of children with a temporary feeding tube, commencing as close to tube placement as possible.

## Methods

### Study design

This study was part of a larger research project, investigating management of children with temporary feeding tubes (Syrmis et al., 2024). Ethics approval was obtained from the Children’s Health Queensland Hospital and Health Service, Human Research Ethics Committee (Reference number HREC/18/QRCH/168) and The University of Queensland Human Research Ethics & Integrity Committee (Reference number: 2022/HE000953). Parents understood that their children’s information was included in the audit, and their data were used in this study. Ethics approval also covered survey administration and use of parent information. Identifiable data were removed, anonymised, and stored securely on a software programme Research Electronic Data Capture (REDCap) version 8.0, manufactured and maintained by Vanderbilt University (Harris et al., 2019). This study is reported in line with the Checklist for Reporting of Survey Studies (CROSS) (Sharma et al., 2021).

### Recruitment and survey administration

A digital registry was established to capture details for hospitalised children who required a temporary feeding tube (e.g. nasogastric, nasojejunal, or nasoduodenal) during two periods: between November 1st, 2018, and February 28th, 2019, and between 1st May and 31st July 2019. Inclusion criteria for identifying eligible children for the registry were hospital inpatients who commenced temporary tube feeding during their admission within the defined study periods; 494 children were initially identified. Upon discharge home, parents of these children were sent an invitation letter by postal mail containing a unique access code and website link to complete an online survey. This process is described in the Supplemental Material (Figure S1). One follow-up letter was sent to non-responders. The survey landing webpage contained detailed study information, covering its purpose, procedures, risks, benefits, and confidentiality. Consent was implied by survey completion, with participants having an opportunity to review this information before proceeding. No incentives were provided for survey completion.

### Survey design and content

The survey included 49 questions and contained three main sections (A, B, C). Parents were asked to self-report their role as mother, father, carer/guardian, their cultural and linguistic background and highest level of education completed. Part A included the Depression Anxiety Stress Scale (DASS-21) (Lovibond and Lovibond, 1995). Part B included the Paediatric Assessment Scale for Severe Feeding Problems (PASSFP), Crist et al. (2004), used to assess progression of oral intake in children who require prolonged tube feeding. Part C included questions exploring parents’ satisfaction with temporary feeding tube experience and four open-ended questions (available in the Supplemental Material).

### Quantitative data analysis

Children’s data from their medical records were analysed using descriptive statistics in Microsoft Excel (version 16.0) to calculate percentages and summarise categorical data. Information gathered from medical records included: child’s age at time of tube insertion, duration of temporary feeding tube use, documentation of a tube exit plan, incidence of tube dependency, discharge plan with a temporary feeding tube, reason for tube removal, and any side effects of temporary tube feeding. DASS and PASSFP survey results were entered into Microsoft Excel to calculate totals, medians, and interquartile ranges.

### Qualitative data analysis

Inductive qualitative content analysis was used to interpret four open-ended survey questions based on guidelines described by Graneheim and Lundman (2004). Firstly, responses to the open-ended questions were read and reviewed several times to understand content and to extract meaning units. Condensed meaning units were then developed to capture key elements of parents’ experiences. Each condensed meaning unit was then coded, and similar codes were grouped into sub-categories and categories. These categories were then examined to develop themes across the data. Wording was checked across categories to ensure it was representative. Data dependability was ensured through data collection and analysis processes, alongside reviews conducted by a subject matter expert (C.R). To ensure research validity, participant responses were coded by two authors independently (C.R. & R.P). Differences were discussed and reviewed until agreement was reached and a third researcher was engaged to support consensus (J.M). Quotes from parent participants are used to support themes identified in the results section. To ensure confidentiality, all participant names are pseudonyms.

## Results

### Quantitative Results

#### Parent Characteristics

A total of 47 parents of the 494 children in the larger audit study (Syrmis et al., 2024) completed the online survey, a response rate of 9.5%. Three surveys were excluded because open-ended questions were not answered, resulting in a final sample of 44 surveys, including 39 mothers and 5 fathers. None of these families came from a culturally and linguistically diverse background or identified as Aboriginal or Torres Strait Islander. Over half of the parents (n = 25, 57%) had a bachelor’s degree and lived within the hospital catchment (n = 24, 55%), which was a metropolitan area. Most parents were happy with the care their child with a temporary feeding tube received (see Figure S2 in Supplemental Material). For parents who completed the DASS (n = 44), a majority reported scores within normal range for anxiety (n = 43, 98%), depression (n = 42, 95%), and stress (n = 40, 91%). No differences in DASS scores were observed across different education levels.

#### Child Characteristics

Survey responses represented 44 children, and characteristics of these children were obtained from their medical notes, described in Table 1. All children in this study had nasogastric feeding tubes as their type of temporary feeding tube. Most children had their temporary feeding tube for ≤5 days (n = 25, 57%), and were discharged home without a feeding tube (n = 37, 84%). Tube feeding dependency was not commonly reported in this cohort and only a small number of tube exit plans were written. In total, 36 parents completed the PASSFP, and a median score of 52.5 (IQR 13.8) was reported. Parents of children discharged home with a temporary feeding tube in situ (n = 7) reported lower scores on the PASSFP (median 45, IQR 17.3) than children who were not discharged home with a tube (median 53, IQR 13), suggesting there were more reported feeding difficulties with the children who were discharged home. The most common indication for tube feeding was primarily for inadequate oral intake due to an acute or chronic illness (e.g., bronchiolitis) (n = 30, 68%).

**Table 1.** Characteristics of children.

	n (%)
Age at tube insertion	
<12 months	27 (61)
1 – 5 years	10 (23)
≥6 years	7 (16)
Duration of time the feeding tube was in place	
≤5 days	25 (57)
6 – 10 days	6 (14)
11–19 days	5 (11)
≥20 days	5 (11)
Tube was still in place^a^	3 (7)
Tube exit plan completed^b^	
Yes	13 (30)
No	31 (70)
Discharged home with a feeding tube	
Yes	7 (16)
No	37 (84)
Main reasons for tube removal^c^	
Medically stable	26 (63)
Adequate oral intake	24 (54)
Not clearly documented	10 (13)
Side effects recorded during the period of tube feeding (n = 13)^b^	
Dislodgement/tube re-insertion	11 (85)
Skin irritation	1 (8)
Nausea/vomiting	3 (23)
Oral aversion	2 (15)
Worsening oral feeding skills	2 (15)
Reduced oral intake	1 (8)
Other	5 (39)
Reported tube feeding dependency	
Yes	1 (2)
No	39 (89)
Unknown	4 (9)

^a^The temporary feeding tube was still in place at the end of the study period (greater than 4 months).

^b^Tube exit plan refers to documentation outlining intended timing and criteria for safely ceasing temporary tube feeding.

^c^The numbers do not equal 100% because more than one option could have been chosen.

### Qualitative Results

#### Themes from Open Ended-Questions

Two main themes were identified from the four open-ended questions, 1) navigating the tube feeding journey; and 2) experiences of the health service. The first theme pertained to the experience of tube placement, adjustment, and management. Tube placement could be a highly emotive time for a child and their parents, with parents reporting “*It is distressing to see your child having a NG tube inserted” [Joy, parent of 13-month-old child]* and *“Both my son and I suffered great anxiety when he would vomit out his feeding tube and need it reinserted*. *He developed significant procedural anxiety with this*. *My son hated getting tape replaced on his face.” [Lisa, parent of an 8-year-old child]*. A few participants noted that it took time for their child to adjust and “*…get familiar with the feeding tube” [Janet, parent of an 8-month-old child]*. One participant commented on needing resilience in adjusting to the tube, reporting that “*your child will most likely hate the tube but with patience, you’ll help them manage the discomfort [Diane, parent of a 6-month-old child]*. Parents required perseverance adjusting to the tube, *“Persistence is key, as is distraction when the tube is first inserted*. *Your child will eventually get used to the feeling of it being there” [Olivia, parent of an 11-month-old child]*.

There were mixed perspectives from participants on managing tube feeding itself. Participants with children that had a temporary feeding tube as an inpatient seemed to adjust well, *“it was easy… we didn’t experience any difficulties with it” [Renee, parent of a 9-month-old child]* when the *“management was handled by the nurses*” *[Olivia, parent of an 11-month-old child].* Those parents of children who were discharged home with temporary tubes described more challenges: *“Management at home was just a lot of trial and error*. *Being new parents we weren't sure what we were doing and then we had some extra difficulties with the pump and medications*. *It was just a matter of working out how it was going to work for our family” [Janet, parent of an 8-month-old child]*. Several participants experienced the impact of the tube when going out in public and experiencing *“peoples curiosity about the feeding tube and having to tell his story a million times.” [Sandra, parent of 1-month-old child]*. Equally, the lack of peer support was noted, with one participant reflecting on their experience, *“I didn’t know anyone else who had been through this*. *I think hearing from other parents might have helped*.*”* *[Anna, parent of a 2-year-old child].*

Many participants acknowledged that the tube was necessary for their child, and it was “…*a relief and allowed her time to grow without the danger of choking” [Serena, parent of a 4-year-old child]*. One parent worried about the impact the tube had on their child’s oral intake, *“I had a lot of anxiety around getting him eating/ drinking/ breastfeeding again and tube feeding dependency” [Rachel, parent of a 10-month-old child]*. Nevertheless, the feeding tube provided comfort to families, fulfilling its intended purpose *“At the time she wasn't eating or drinking so having a tube feed in was a comfort to know she was getting the nutrition she needed” [Casey, parent of a 1-year-old child]*.

The second theme described participants’ experiences of the health service. A majority of participants expressed that they felt empowered by the health service regarding their child’s temporary feeding tube. Those that felt empowered reported more positive experiences, acknowledging that “*It was helpful to have nurses who kept my partner & I well informed about the tube” [Diane, parent of a 6-month-old-child] and “involved us in the decision making” [Glenn, parent of a 7-month-old child]*.*”* Some participants described more negative experiences; one parent shared that *“Staff re-assured [the child] “that it [the tube] would be slightly uncomfortable during insertion”, when in practice it was horrendously painful resulting in crying for some time”*. *[Brad, parent of a 14-year-old child]*. Several participants commented that communication from and among health professionals was inconsistent and confusing, reporting *“It is also difficult when her medical team has contradictory opinions about the best way to manage growth/feeding issues” [Shay, parent of a 4-year-old child]*.

However, a vast majority of participants indicated that they had confidence in their health professionals and trusted the care they provided, reporting that they “...*do their best to keep our children alive and help them get better*” *[Kate, *parent* of 2-month-old child],* believing that their child was *“in safe hands” [Vivian, parent of a 3-year-old child]*. Many parents wanted to learn how to independently manage their child’s temporary feeding tube. Positive experiences of health professionals were reported when parents were involved and educated in tube management, with one parent stating: “*they [health professionals] are good teachers when learning how to use the machines and how to put in the tubes” [Sandra, parent of 1-month-old child]*. Parents’ involvement was driven by their desire to actively understand and engage in their child’s care and be *“confident to use it [the tube] at home without assistance” [Jen, parent of a 1-year-old child]*. Several participants were disappointed, however reporting that *“it would have been helpful to have been offered a few more instructions and opportunities to practice using the tube while in hospital” [Brad, parent of a 14-year-old child],* appreciating that this knowledge *“helped develop skills and confidence for home” [Lisa, parent of an 8-year-old child]*. In general, the responsiveness of health professionals was appreciated by participants, with one participant describing that *“all the support has been helpful*. *When questions were posed, we generally received timeous and helpful feedback” [Chris, parent of a 15-year-old child]*.

Many participants wanted to be involved in decision-making relating to their child’s feeding tube, and advocacy for involvement in the decision making was identified as important by several participants, with one parent encouraging others to *“always ask questions if you are unsure and don’t be scared to voice your opinion” [Anna, parent of a 2-year-old child]*.

In response to a survey question, participants shared advice for other parents, reflecting both their informal expertise and a desire to support others facing similar challenges. Their advice focused on managing tube insertion distress, coping with daily life, building tube care confidence, and advocating within health professionals (see Supplemental Material).

## Discussion

This study aimed to describe experiences of parents of children with a temporary feeding tube, by exploring parental insights via a web-based survey. Unlike previous research that does not differentiate types of feeding tubes (Pahsini et al., 2016; Pedersen et al., 2004; Wilken, 2012), this study uniquely addresses the specific experiences of parents of children with temporary feeding tubes.

Consistent with prior research from Australia, Canada, and Europe (Cipolla et al., 2022; Lively et al., 2023; Remijn et al., 2022; Serjeant and Tighe, 2022), parents in this study emphasised the importance of active involvement and participation in decision-making regarding their child’s temporary tube feeding care. Parent participants similarly highlighted confusion and stress arising from inconsistent advice provided by their health professionals. This reinforces the critical need for coordinated communication and consistent reliable information. Although focused on children with gastrostomy tubes, studies by Murphy et al. (2023) and Suluhan et al. (2021) describe similar parental experiences, suggesting that coordinated care and family support are likely just as essential in temporary tube feeding contexts.

These findings provide a unique insight into challenges faced by parents of children with temporary feeding tubes after discharge home from hospital. An important finding of this work was that families experienced substantial challenges at home, struggling with completing tube care independently, and described information gaps, stigma, and increased stress. Few children had documented tube exit plans in their medical notes, despite recommendations for their completion at the time of the tube insertion to facilitate a timely tube removal and prevent tube dependency (Dunitz-Scheer et al., 2009). These experiences align with previous research on temporary tube feeding, which identified inadequate preparation, and education during the transition from hospital to home (Culverwell, 2005; Mekhuri et al., 2025). One parent in this study identified a lack of peer support as a significant gap, expressing that connections with other families could have been helpful, consistent with previous recommendations (Dodds and Walch, 2022; Jose et al., 2021; Kinsella et al., 2024). These findings reinforce the importance of thorough discharge planning to better prepare families for care at home (Khair, 2003).

This study demonstrated that families received limited psychosocial and emotional support. While the majority of communication from health professionals was reported to be positive by parents, the nature of this communication appeared to be medical rather than psychosocial. Parents frequently reported feeling empowered and valued when involved in decision-making and explained practical tube-related information. However, whilst not specifically asked in the survey, no parents mentioned receiving targeted emotional or psychosocial support. This finding aligns with previous research suggesting health professionals focus on medical aspects of tube feeding care (Brotherton and Abbott, 2009; Remijn et al., 2022; Serjeant and Tighe, 2022). With previous studies documenting substantial psychosocial impacts for parents caring for children with feeding tubes (Banhara et al., 2020; Rollins, 2006), exploration into targeted emotional support interventions is recommended, particularly for temporary feeding tubes where families may receive less comprehensive discharge planning due to assumptions regarding reduced burden and short-term requirements.

The distress associated with tube insertions was highlighted in the findings of this study. Parents reported that tube insertions were distressing both for children to endure and for themselves to witness, in line with prior research findings (Babl et al., 2009, 2012; Crellin and Johnston, 2005; Ferguson and Campbell, 2014; Krom et al., 2019; Remijn et al., 2022; Serjeant and Tighe, 2022). However, the longer-term psychological impact of this distressing experience remains unexplored. Prior research has demonstrated that approximately 30% of parents developed post-traumatic stress disorder (PTSD) following their child’s medical procedure (Burgess, 2019). This question warrants further investigation for temporary feeding tubes, where frequent re-insertions may compound family stress and trauma.

### Limitations

This study has provided some new insights regarding parents’ experiences of temporary feeding tubes; however, several limitations are acknowledged. The response rate for this survey was low (9.5%), and there was limited diversity across different cultural, socioeconomic, and demographic groups, with data collected from one facility. Therefore, findings may not be representative of all parents of children with temporary feeding tubes. Given this low response rate, there is a potential for volunteer bias, meaning the experiences reported may not fully represent the spectrum of all parents, possibly skewing towards those with either more positive or negative experiences who were more inclined to participate (Mazor et al., 2002). It is also acknowledged that parents with lived experience were not involved in the survey design, and, as a result, questions may not have fully captured the authentic experiences of this cohort. Including health consumers (parents with lived experiences of tube feeding a child) in the study design may have improved the survey instrument, response rate, and participant diversity. Additionally, the survey did not include questions specifically about parents’ experiences at home, limiting understanding for these families. Finally, because tube placement was often short-term for this cohort, many parents completed this survey after their child’s feeding tube was removed, and their responses may have been different if they completed the survey when their child had their tube inserted.

### Implications for practice

Findings of this study have implications for the clinical practice of health professionals managing children with temporary feeding tubes. It is suggested that health professionals incorporate structured educational sessions for parents as part of the temporary tube feeding management process. These sessions could cover not only technical aspects of tube maintenance but also strategies for managing psychological, social, and pain-related challenges associated with temporary tube feeding after discharge home. Additionally, healthcare facilities could consider implementing a standardised communication protocol that ensures all health professionals provide consistent and clear guidance to families. This could involve protocolised medical notes to align treatment approaches and progress, thus minimising inconsistencies in information relayed to families. To further support families, hospitals could develop a comprehensive discharge plan checklist that includes training, support resources, pain management strategies, and hospital contact details.

## Conclusion

This is one of the first studies to intentionally capture the experiences of parents of children with temporary feeding tubes, making it an important but preliminary contribution to our understanding of temporary tube feeding journeys, from tube placement to removal. The findings from this study indicate that parents need to feel empowered through effective communication and inclusion in decision-making throughout their child’s temporary tube feeding journey. Future research should focus on interventions that support families throughout their tube feeding journey, especially for those with children discharged home with a temporary feeding tube. By understanding and addressing specific needs of families with children on temporary feeding tubes, healthcare professionals can improve both the immediate and long-term outcomes for these children and their families.

## Supplemental Material

Supplemental Material - Experiences and insights from parents of children with temporary feeding tubes Supplemental Material for Experiences and insights from parents of children with temporary feeding tubes by Claire Reilly, Jeanne Marshall, Rebecca Packer, Nikhil Thapar, Maryanne Syrmis, Nadine Frederiksen and Kristie L Bell in Journal of Child Health Care
