# Supplementary material for: Experiences and insights from parents of children with temporary feeding tubes
Source: J Child Health Care. 2025 Aug 23;30(3):446–57. doi: 10.1177/13674935251370415 (PMC13490679; doi:10.1177/13674935251370415)
Supplement: Supplementary file 1 [file sj-zip-1-chc-10.1177_13674935251370415.zip › Description of Supplementary Material.pdf]

Description of Supplementary Material

Table S1. Parent-to-parent advice on managing temporary nasogastric tube feeding

Caption: Verbatim responses from parents who completed the survey question providing advice for others.

Figure S1. Flow diagram of the parent survey process.

Caption: Depicts eligibility, exclusions, and final number of parent survey responses included in the analysis.

Figure S2. Parent ratings of hospital support and satisfaction with tube feeding management.

Caption: Responses to two survey items using a 5-point Likert scale from 0 (“Very Low”) to 4 (“Very High”).

Supplementary Material. Part C of the Survey.

Caption: Survey items related to parent satisfaction, perceived impact of tube feeding, hospital support, decision-making, and coping strategies.
