# Supplementary material for: Experiences and insights from parents of children with temporary feeding tubes
Source: J Child Health Care. 2025 Aug 23;30(3):446–57. doi: 10.1177/13674935251370415 (PMC13490679; doi:10.1177/13674935251370415)
Supplement: Supplementary file 1 [file sj-zip-1-chc-10.1177_13674935251370415.zip › Supplementary material_Part C of the survey.pdf]

Part C: Satisfaction with Nasogastric (NGt) Feeding

How do you rate your satisfaction with your child's NGt feeding management (select one number on the scale)

0 - Very low	1	2 – Neutral	3	4 – Very High

How do you perceive change in your child's nutritional status as a result of NGt feeding?

0 - Very low	1	2 – Neutral	3	4 – Very High

Please describe what is has been like for you and your family in managing your child's feeding/eating difficulties and NGT management?

What are some of the things that have helped you to cope/manage better?

How do you rate the support offered from the hospital regarding your child's tube feeding?

0 - Very low	1	2 – Neutral	3	4 – Very High

Were you involved in the decision for your child to have a NGT? Yes/ No

Did you receive enough information before the NGT was inserted? Yes/ No

Please identify how the support offered by the hospital has been helpful or not helpful:

What advice would you give to other parents who are in the same situation as you, or about to embark upon the same experience as you?
