# Supplementary material for: Experiences and insights from parents of children with temporary feeding tubes
Source: J Child Health Care. 2025 Aug 23;30(3):446–57. doi: 10.1177/13674935251370415 (PMC13490679; doi:10.1177/13674935251370415)
Supplement: Supplementary file 1 [file sj-zip-1-chc-10.1177_13674935251370415.zip › Table S1_Advice given by parents.pdf]

Table S1. Parent-to-parent advice on managing temporary nasogastric tube feeding

Theme	Advice given by parents (parent role and age of child)
**Reassurance about tube insertion & adjustment**	"That your child will get quite upset but that once the process is over they settle down and they generally don't notice that it's there." (mother of a 12-month-old child)<br>"It is distressing to see your child having a NG tube inserted, so keep bearing in mind that it's for the best interests of your child." (mother of a 13-month-old child)<br>"Persistence is key, as is distraction when the tube is first inserted. Your child will eventually get used to the feeling of it being there." (mother of an 11-month-old child)<br>"Your child will most likely hate the tube but with patience, you'll help them manage the discomfort." (mother of a 6-month-old infant)<br>"Don't be scared or worried. At first it is a bit scary but once you have training how to feed your baby and test the tube it really does become part of everyday routine." (mother of a 1-month-old infant)<br>"Don't be scared by the tube like I was… Learn about it. Be involved at the hospital with it so it isn't so scary when you are home." (mother of a 12-month-old child)
**Coping in day-to-day life & public settings**	"Not being scared to get out of the house, even if people stare or ask questions." (mother of a 8-month old child)<br>"Just ignore the tube. It looks 100 times worse than what it is." (mother of a 2-month old child)
**Advocating & interacting with clinicians**	"Always ask questions if you are unsure and don't be scared to voice your opinion." (mother of a 2-year-old child)<br>"Ask the treating team how an intervention or change is specifically directed towards achieving a specific outcome, not just default practice." (mother of a 1-month-old infant)<br>"Insist on the use of a child-sized feeding tube… Be aware it may be painful but is necessary, and removal/re-insertion can occur if treatment is refused." (father of a 14-year-old adolescent)
**Deciding on tube use**	"If your child needs a tube for hospitalisation, do it." (father of a 2-month-old infant)
